# Uptake of COVID-19 Vaccination and Related Factors Among People who Inject Drugs, San Francisco, 2022

**DOI:** 10.1007/s10461-024-04564-z

**Published:** 2024-12-10

**Authors:** Said Iftekhar Sadaat, Alexander Marr, Ali Mirzazadeh, Bow Suprasert, Moranda Tate, Erin Wilson, Willi McFarland

**Affiliations:** 1 Institute for Global Health Sciences, University of California San Francisco, 550 16th St, San Francisco, San Francisco, CA 94518 USA; 2 Center for Public Health Research, San Francisco Department of Public Health, CA, San Francisco USA; 3 Department of Epidemiology and Biostatistics, Institute for Global Health Sciences, University of California San Francisco, CA, San Francisco USA

**Keywords:** COVID-19 Vaccination, People who inject drugs, Homelessness

## Abstract

We assessed the uptake of COVID-19 vaccination in a community-recruited sample of people who inject drugs (PWID) in San Francisco in 2022. Overall, 72.4% (95% CI 64.6–80.3) were vaccinated for COVID-19. Independent predictors of vaccination were age 65 years and older (adjusted odds ratio [AOR] 9.7, 95% CI 2.2–28.7) and ever testing positive for COVID-19 (AOR 2.0, 95% CI 1.2–3.5). Homelessness was associated with lower COVID-19 vaccination (AOR 0.5, 95% CI 0.3–0.8). Our study underscores the urgent need for targeted interventions to address unique challenges faced by PWID in accessing COVID-19 vaccination, particularly for those experiencing homelessness and who are younger.

## Introduction

The Centers for Disease Control and Prevention (CDC) recommends staying up to date with COVID-19 vaccinations as they are effective, safe, and free in the United States [1]. COVID-19 vaccines were shown to be highly effective both in clinical trials and in real life in reducing the transmission of disease and preventing severe illness and mortality. A meta-analysis of the effectiveness of several COVID-19 vaccines, including AstraZeneca, Pfizer, Moderna, Bharat, and Johnson & Johnson, reported that after the first dose, the total effectiveness of all COVID-19 vaccines was 71% (95% CI 0.65–0.78) while the total effectiveness after the second dose was 91% (95% CI 0.88–0.94) [2]. However, the benefits of COVID-19 vaccines depend on acceptance and coverage among various populations.

The national coverage for at least one dose of COVID-19 vaccine among adults 18 years and older in the general population as of February 2024 was 81.5% (95% CI 80.7–82.2) in the United States, where it was 84.4% (80.8–87.9%) in California and 95.7% (95.65–95.74%) in San Francisco [3]. However, COVID-19 vaccination coverage has been low among people who inject drugs (PWID) in comparison to the general population. A community-based cohort study of PWID in San Diego, California, conducted from October 2020 to September 2021, reported that only 37.8% of PWID received at least one dose of COVID-19 vaccine [4]. PWID participating in a cohort study in Baltimore, Maryland, reported that 68% of PWID had received at least one dose of COVID-19 vaccine by September 2021 [4]. Another cohort study of PWID in Baltimore found that by February 2022, 70% of PWID in Baltimore were vaccinated with at least one dose of COVID-19 vaccines [5]. Another study using respondent-driven sampling (RDS) conducted from 2021 to 2023 among PWID in New York found that around 80% received at least one COVID-19 vaccine dose [6]. Vaccine uptake among PWID also has been low in other countries. For example, the Australian Needle Syringe Program Survey in 2021 reported that only 49% of the PWID received at least one COVID-19 vaccine dose, which was significantly lower than the general population [7].

PWID are particularly at risk for COVID-19 and may suffer more severe disease than the general population [8]. The population has had low healthcare usage due to reasons such as insufficient health insurance, transportation challenges, stigma, and distrust in the medical system [4]. In addition, the PWID might have various underlying health issues such as respiratory or pulmonary conditions, compromised immunity, or chronic liver disease, which increase their risk of severe outcomes from COVID-19 [7]. Therefore, PWID needs to be prioritized for targeted interventions for primary and boosting COVID-19 vaccination.

We examined COVID-19 vaccine uptake in a community-based sample of PWID in San Francisco in 2022 and identified factors associated with vaccine uptake. We also assessed individual (demographic characteristics, employment, living area, sexual identity) and structural (health insurance, incarceration, homelessness) factors associated with COVID-19 vaccination.

## Methods

Our study data are from the 2022 cycle of the National HIV Behavioural Surveillance (NHBS) conducted in San Francisco from June to December 2022 [9] as part of the CDC’s National HIV Behavioural Surveillance system [10]. The NHBS includes serial cross-sectional surveys of PWID conducted nationally in 20–23 metropolitan statistical areas [10] for monitoring the prevalence of HIV infection and its related behaviors among groups who are at high risk of acquiring HIV infection [9].

In the NHBS 2022, the participants were recruited through RDS (Respondent-Driven Sampling), a sampling methodology through the recruitment of seeds and long chains of peer referrals among hard-to-reach populations [10]. Initially, ten seeds who were eligible PWID from diverse social networks were enrolled and instructed to recruit three to five eligible PWID from their social networks. These recruits also referred other eligible PWID from their networks until achieving the intended sample size of 500 [11]. In order for the subjects to be recruited for the study, the criteria were 18 years or older, speaking English or Spanish, residing in San Francisco or San Mateo counties, and self-reported injecting drug use in the last 12 months [9]. Incentives were $75 for completing the study and $10 for each eligible peer referred enrolled. The Internal Review Board of the University of California San Francisco reviewed and approved the NHBS (IRB#19–29460). Participants provided verbal informed consent to preserve anonymity [11]. To comply with the COVID-19 prevention policies, the interviews were conducted on-site via nearby videoconference instead of in-person, face-to-face interviews as in previous cycles of NHBS [9].

Our outcome is the answer to the broad question: “Have you been vaccinated for COVID-19?” We report crude and RDS-adjusted estimates. To produce sampling weights, we used the RDS package in R software and Gile’s successive sampling estimator [10]. We measured the coverage of COVID-19 vaccination overall and by subgroups of age, gender, marital status, education, employment, sexual identity, homelessness, health insurance, HIV status, HCV testing, ever positive for COVID-19, and zip code. We first tested for significant differences in COVID-19 vaccination sub-group categories of each variable using the Chi-square test with variance adjustments for the RDS sampling design. We then assessed independent associations between predictors and COVID-19 vaccination using multivariable logistic regression analysis as previously reported [10]. We included all variables with a p-value of less than 0.2 in bivariate comparisons as candidate variables. We considered p-values < 0.05 significant and retained them in the final multivariable model.

## Results

### Sample Characteristics

The majority of participants were older than 40 years (74.6%), male (67.7%), not cohabitating with a partner (91.4%), had secondary education or less (57.7%), were unemployed (64.9%), had health insurance (91.8%), and identified as heterosexual/straight (73.1%) (Table 1). Two-thirds (67.5%) resided in the two zip codes that make the inner-city neighborhoods of the Tenderloin and South of Market neighborhoods, with two-thirds (66.7%) experiencing homelessness in the last year. The sample was diverse with respect to race/ethnicity. Having tested HIV positive was reported by 9.6 % . Testing positive for COVID-19 was reported by 79.2 % .

**Table 1:** Uptake of COVID-19 vaccine among people who inject drugs in San Francisco, NHBS^1^, 2022 (N = 527)

Characteristics	Sample n (col. %)	Crude vaccinated n (row %)	RDS^2^-adjusted row % vaccinated (95% CI)	Test statistic	p-value^3^	Adjusted OR (95% CI)	p-value^4^
Total	527	370 (70.2)	72.4 (64.6–80.3)		–	–	–
Age group (years)							
22–29	25 (4.7)	11 (44.0)	67.7 (33.9–89.6)	X^2^ = 26.20	0.057	1	
30–39	109 (20.7)	64 (58.7)	75.6 (64.0–84.3)			1.9 (0.8–4.7)	0.154
40–49	146 (27.7)	95 (65.5)	58.9 (42.9–73.1)			2.6 (1.1–6.5)	0.030
50–59	166 (31.5)	129 (78.2)	74.7 (59.3–85.7)			4.6 (1.9–11.4)	0.001
60+	81 (15.4)	71 (88.7)	88.1 (71.7–95.5)			9.7 (2.2–28.7)	0.0001
Gender							
Male	357 (67.7)	255 (72.0)	72.9 (63.3–80.8)	X^2^ = 7.01	0.198		
Female	157 (29.8)	103 (65.6)	68.9 (53.8–80.8)				
Transgender	13 (2.5)	12 (92.3)	98.3 (83.0–99.8)				
Partner status							
Not cohabiting	478 (91.4)	341 (60.0)	64.1 (36.5–84.7)	X^2^ = 2.24	0.462		
Cohabiting	45 (8.6)	27 (71.8)	73.4 (65.1–80.3)				
Education							
Secondary or less	303 (57.7)	222 (73.5)	78.6 (69.0–85.9)	X^2^ = 12.79	0.056		
More than secondary	222 (42.3)	147 (66.8)	64.6 (51.7–75.6)				
Employment							
Unemployed	336 (64.9)	216 (64.9)	66.6 (56.5–75.4)	X^2^ = 13.07	0.223		
Unable to work	118 (22.8)	97 (82.2)	80.8 (64.1–90.9)				
Employed	64 (12.4)	52 (81.2)	81.7 (52.8–94.7)				
Current health insurance							
Not insured	43 (8.2)	26 (60.5)	70.4 (44.9–87.5)	X^2^ = 0.06	0.856		
Insured	484 (91.8)	344 (71.5)	72.5 (64.3–79.5)				
Sexual orientation							
Heterosexual	380 (73.1)	259 (68.5)	69.5 (59.8–77.8)	X^2^ = 5.37	0.440		
Homosexual	34 (6.5)	26 (78.8)	82.6 (54.3–94.9)				
Bisexual	106 (20.4)	80 (75.5)	77.9 (59.2–89.6)				
Zip code							
All other zip codes	169 (32.5)	121 (72.0)	80.6 (71.5–87.3)	X^2^ = 6.65	0.076		
Inner city zip codes	351 (67.5)	245 (70.0)	69.6 (59.1–78.4)				
Homeless last year							
No	175 (33.3)	142 (81.6)	81.6 (69.3–89.7)	X^2^ = 12.92	0.060	1	
Yes	351 (66.7)	228 (65.3)	67.5 (57.3–76.3)			0.5 (0.3–0.8)	0.003
Race/ethnicity							
Black	166 (31.8)	137 (83.0)	80.1 (64.1–90.1)	X^2^ = 10.87	0.565		
White	246 (47.1)	152 (62.0)	68.9 (58.4–77.8)				
Hispanic	63 (12.1)	46 (73.0)	76.1(50.3–90.9)				
Other	15 (2.9)	11 (73.3)	64.4 (26.6–90.0)				
Multiple	32 (6.1)	22 (68.7)	61.0 (28.9–85.7)				
Incarceration							
Not held or arrested past 12 months	417 (79.1)	302 (72.8)	70.4 (61.2–78.3)	X^2^ = 4.43	0.111		
Held or arrested past 12 months	110 (20.9)	68 (62.4)	80.9 (70.1–88.5)				
Ever tested positive for HIV							
No	451 (90.4)	310 (69.2)	70.8 (62.2–78.1)	X^2^ = 3.87	0.417		
Yes	48 (9.6)	42 (87.5)	82.8 (47.2–96.3)				
Ever tested for HCV							
No	70 (13.3)	52 (74.3)	79.5 (64.2–89.4)	X^2^ = 1.89	0.310		
Yes	457 (86.7)	318 (70.0)	71.4 (62.7–78.8)				
Ever tested positive for COVID-19							
No	415 (79.7)	284 (68.4)	69.0 (59.8–76.9)	X^2^ = 12.53	0.015	1	0.009
Yes	106 (20.4)	84 (79.2)	86.6 (74.4–93.5)			2.0 (1.2–3.5)	

1. National HIV behavioral surveillance

2. Respondent driven sampling

3. P-values and chi-square statistic by chi-square test on variance-adjusted estimates

4. P-values by multivariable logistic regression analysis

### COVID-19 Vaccine Uptake

Table 1 shows the sample (crude) and Respondent Driven Sampling (RDS)-adjusted prevalence of COVID-19 vaccination history. Overall, COVID-19 vaccination coverage among PWID in San Francisco was estimated at 72.4% (95% CI 64.6–80.3). Although vaccination coverage did not vary significantly across many characteristics, including gender, cohabitation, employment, insurance, sexual orientation, race/ethnicity, incarceration, HIV status, and ever-tested for HCV, some notable differences were observed. For example, vaccination rates by racial group showed that Black individuals had a higher coverage of 80.1% vs. 68.9% for White and 76.1% for Hispanic individuals (χ²= 10.87; *p* = 0.565). In a similar instance, the vaccination rate was 80.6% in areas classified as “all other zip codes” in San Francisco, while it was 69.6% in inner-city zip codes (χ²=6.65; *p* = 0.0.076). Likewise, individuals who were held or arrested in the past 12 months exhibited a vaccination rate of 80.9% vs. 70.4% for those who were not held or arrested in the past 12 months ((χ²= 4.43; *p* = 0.111). The bivariate analysis found borderline (*p* > 0.05 but < 0.10) trends for higher vaccination coverage among older age groups, those with lower education levels, those with non-inner-city zip codes, and those experiencing homelessness. Testing positive for COVID-19 was significantly associated with higher vaccination coverage. In multivariate analysis, two factors were significantly associated with higher COVID-19 vaccination coverage among PWID. First, compared to the youngest age group (22–29 years old), those aged 40–49 years (adjusted odds ratio [AOR] 2.6, 95% confidence interval [CI] 1.1–6.5), aged 50–59 years (AOR 4.6, 95% CI 1.9–11.4), and 60 years and older (AOR 9.7, 95% CI 2.2–28.7) had higher vaccination coverage. Second, having tested positive for COVID-19 was associated with higher vaccination compared to those who never tested positive (AOR 2.0, 95% CI 1.2–3.5). Homelessness in last year was significantly associated with lower odds of vaccination (AOR 0.5, 95% CI 0.3–0.8).

## Discussion

Our study found that nearly three-fourths of PWID surveyed had been vaccinated for COVID-19 within the two years of the vaccine’s first availability. Coverage increased with increasing age, nearly achieving 90% among those aged 60 years or more. However, PWIDs who faced homelessness in the last year had half the odds of being vaccinated. These findings point to the need for programs to specifically reach younger PWID and those experiencing homelessness.

Our data place COVID-19 vaccination coverage in San Francisco among PWID lower than the general population of California (84.4%) and substantially lower than the general population of San Francisco (95.7%) [3]. Nonetheless, vaccination coverage was still higher than the uptake among PWID in other cities in the United States. For example, the coverage for at least one dose of COVID-19 vaccination among PWID was estimated at 10% in Oregon, 38% in San Diego, and 68% in Baltimore [4]. Efforts in San Francisco to improve vaccine access included offering vaccination at multiple locations in health facilities and through “street medicine outreach” at encampments and other areas of high drug activity. In a study of barriers and enablers to COVID-19 vaccination in San Francisco [12], more than half of the participants referred to the encouraging and supportive roles of community-based organizations and several community leaders (e.g., church leaders and neighbours involved in non-profit organizations). Several participants reported other enablers such as navigators to vaccine sites, outreach from clinics, language and cultural concordance of information, and a diversity of vaccination sites.

Nonetheless, vaccination was lower for the PWID population compared to the general population of San Francisco. The reduced COVID-19 vaccination rates among PWID increase their risk of illness and death from COVID-19. Moreover, there was a substantial gap in reaching the PWID population experiencing homelessness. Previous research suggests that PWID populations may experience multiple barriers to vaccination, including individual barriers (e.g., poor or misunderstanding of safety or effectiveness of vaccines) and structural barriers (e.g., lack of clear communication, service navigation, access, and linkage services) [12].

We acknowledge the limitations of our study. First, we measured the COVID-19 vaccination status by self-report, not by assessing antibodies or medical records. Moreover, we only broadly asked if they were vaccinated without specifying how many doses or what type of vaccine. Second, our results may not be generalizable beyond San Francisco, especially given our city’s high overall level of vaccine uptake. Third, we did not measure other important variables, such as co-morbidities associated with COVID-19 morbidity and mortality. Therefore, we were not able to assess the vaccine coverage among subpopulations who most needed the vaccine. We also note the limitation of not being able to establish cause before effect. This particularly affects the interpretation of having a positive COVID-19 test result being associated with being vaccinated. We believe that persons previously experiencing the symptoms of COVID-19 may be more likely to seek vaccination as the explanation. Longitudinal studies and clinical trials are more appropriate for interpreting protection from subsequent infection.

In summary, our study identified substantial gaps in COVID-19 vaccination among PWID in a city that had high overall coverage. More efforts are needed to increase coverage of COVID-19 vaccination among PWID, especially those experiencing homelessness and those who are younger.
